# Impact of coccidial infection on vaccine- and vvIBDV in lymphoid tissues of SPF chickens as detected by RT-PCR

**DOI:** 10.1186/1751-0147-48-17

**Published:** 2006-09-04

**Authors:** Susanne Kabell, Kurt J Handberg, Magne Bisgaard

**Affiliations:** 1 Danish Institute for Food and Veterinary Research, Hangøvej 2, DK-8200 Aarhus N, Denmark; 2 Department of Veterinary Pathobiology, The Royal Veterinary and Agricultural University, 4 Stigbøjlen, DK-1870 Frederiksberg C, Denmark

## Abstract

**Background:**

This study aimed at investigating a potential effect caused by coccidia on the immune response to vaccine- and very virulent infectious bursal disase virus (vvIBDV) in SPF chickens.

**Methods:**

Two groups of three weeks old SPF chickens were vaccinated prior to inoculation with coccidia and challenge with virulent IBDV, all within a period of eight days. Two control groups were similarly treated, except that challenge with field virus was omitted in one group while inoculation with coccidia was omitted in the other group. Clinical signs, lesions in the intestines caused by coccidia, lesions in the bursa of Fabricius caused by IBDV, IBDV-antibody titres, and virus detection by reverse transcription polymerase chain reaction (RT-PCR) were compared among the groups. Lymphoid tissues and swab samples were analysed by general RT-PCR, and positive results were identified by strain specific duplex (DPX) RT-PCR.

**Results:**

In the tripple-infected groups, vaccine strain IBDV was detected in spleen and thymus tissues, and no field virus was detected in bursa samples, contrary to the double-infected groups.

**Conclusion:**

The results suggest an enhancing effect on the immune response caused by subclinical coccidiosis and vvIBDV acting in concert.

## Background

Gumboro disease, subsequently named infectious bursal disease (IBD) is a disease in young chickens caused by infectious bursal disease virus (IBDV), a double stranded, bi-segmented RNA virus [1]. Two serotypes, 1 and 2, have been reported, serotype 1 being the only one pathogenic to the domestic chicken [2]. Several serotype 1 strains of varying virulence have emerged, as reviewed by [3]. So far definitive virulence markers have not been identified. IBDV targets the IgM positive B-lymphocytes in the bursa of Fabricius [4,5], transiently compromising the humoral as well as the cellular immune responses [6,7].

Infection with virulent IBDV in three to six weeks old chickens causes high morbidity and mortality, followed by immunosuppression in surviving chickens [8]. Exposure at an earlier age only results in immunosuppression [9]. The immunosuppression may cause increased susceptibility to different antigens including *Salmonella *[10], infectious bronchitis [11], Newcastle disease [12] and *E. tenella *[13,14]. However, the reverse situation, the impact of different infections on the course of an IBDV infection, vaccination included, remains to be investigated.

Problems due to IBDV outbreaks have been reported from Denmark [15] and several other countries [16-18] even in vaccinated flocks [19], raising speculations that various stress factors, subclinical infections in particular, might influence the outcome of vaccinations. *Eimeria *species are regarded as ubiquitous parasites in most poultry environments, colonizing chicken guts after oral uptake of sporulated oocysts. Coccidial infections are traditionally controlled by coccidiostats in the feed. However, in Denmark whole wheat is gradually added to the feed, and from the age of three weeks and until processing, 25–30% of the broiler feed may be substituted by wheat. The concentration of coccidiostats decreases proportionally, increasing the risk of subclinical coccidiosis. *E. tenella *mainly replicates in the epithelium of the cecae, but developing stages of *E. tenella *have been found in the bursa of Fabricius [13], also involved in replication of IBDV [20]. Development of protective immunity towards coccidia mainly includes the cell-mediated immune system [21], while protection against IBDV, previously assumed based on the humoral immune response [22], also depends on T-cell involvement [23]. Indeed, Yeh *et al*. [24] have reported, that chemically bursectomised chickens re-infected with IBDV, in the absence of humoral antibodies against IBDV were protected by the cell-mediated immune system alone. As coccidia and IBDV may target the same age group of chickens and invade the same tissue, and immunity also to some extent relies on similar factors, we aimed at investigating the influence of a subclinical coccidial infection on IBD vaccinated chickens, subsequently challenged with vvIBDV. The objective of our study was investigation of the effect of the presence of coccidia on the tissue distribution of vaccine and vvIBDV. In a previous paper, we documented indications of vvIBDV replication in lymphoid tissues of vaccinated chickens [25]. We suspected that subclinical coccidial infection might stress the chickens enough to aggravate field strain replication in spite of vaccination.

Parameters investigated and compared included clinical signs, pathological lesions in the intestines and in the bursa of Fabricius, seroconversion, and presence of viral RNA in lymphoid tissues and bursal swab samples.

## Methods

### Chickens

SPF eggs from Lohmann Tierzucht (Cuxhaven, Germany) were hatched under laboratory conditions, and chickens were reared as described previously [26]. The method for euthanisation of chickens was in accordance with Article 2(1) in Directive 86/609/EEC of 24 November 1986.

### Virus

Virus strains used included the commercially available IBD vaccine strain D78 and the virulent strain DK01, previously described [26].

### Coccidial strain and inoculation protocol

*E. tenella *oocysts from a Swedish field outbreak were kindly provided by Dr. P. Thebo, S.V.A., Uppsala. The oocysts were sporulated and kept in 2% potassium dichromate solution at 12°C [27]. The solution contained 200 000 sporulated *E. tenella *oocysts per ml and approximately 10 *E. acervulina *oocysts per ml, as estimated by counting in a McMaster chamber under a microscope. The dose of sporulated oocysts was adjusted to infect the birds without causing mortality [28]. The optimal dose was decided according to results of the following preliminary experiment: Twelve chickens were vaccinated with D78 according to vaccine company recommendations (Intervet, Boxmeere, The Netherlands) when they were 21-days old. At day 24 they were divided into four groups of three chickens each and marked by leg marks. After a feed withdrawal period of four hours, sporulated *E. tenella *oocysts were given orally in the following doses, 0, 150, 1500 and 15 000. The chickens were euthanised seven days after inoculation, and autopsy was performed immediately to evaluate the degree of coccidial infection. Lesion score results, evaluated as described under detection of coccidia, were as follows: 0,0,0; 0,0,0; 3,2,2 and 3,3,3. From these results it was decided to inoculate three experimental groups with 1500 sporulated oocysts each, in order to mimic subclinical coccidiosis.

### Detection of coccidia

The intestinal tract was removed immediately after death and examined macroscopically under strong light. Lesion scores were evaluated according to *Johnson & Reid *[29]. Concerning *E. tenella*, 0 = no gross lesions, 1 = few scattered petechiae on the cecal wall, normal cecal wall and contents, 2 = more numerous petechiae, cecal wall somewhat thickened, blood present in cecal contents, 3 = coalescent petechiae, cecal walls greatly thickened, much blood and fibrin cloths in cecal contents, 4 = Cecal walls greatly swollen and thickened, distended with blood or caseous clots. Concerning *E. acervulina*, 0 = no gross lesions, 1 = few elongated white patches in the duodenal wall in a ladder-like aspect, normal intestinal wall and contents, 2 = numerous white patches in the duodenal wall, normal intestinal wall and contents, 3 = coalescent lesions in the entire duodenum, thickened intestinal wall, contents watery and slimy, 4 = completely coalescent lesions, greyish mucosa, greatly thickened intestinal wall, mucoid contents.

In addition, a smear was prepared from duodenum, jejunum and cecum in order to confirm the presence of coccidia by examination directly under light microscope.

### Histology

Bursa tissues were fixed in 4% formaldehyde overnight, embedded in paraffin wax and cut in 2 μ sections, mounted on Super Frost Plus slides and stained with hematoxylin and eosin (HE) [30].

Lesions observed in bursa tissues were quantitatively transformed. Bursa samples showing no lesions were assigned a value of 0, lesions involving between one and 25% of the follicles was assigned a value of 1, 26 to 50% was assigned a value of 2, 51 to 75% was assigned a value of 3, and 76 to 100% affected follicles was assigned a value of 4.

### Serology

Serum samples were stored at -20°C until analysed. The Infectious Bursal disease Antibody Test Kit 113 from BioChek B.V. (Gouda, The Netherlands) was used as recommended by the manufacturer. Microsoft Exel was used for calculating the IBDV ELISA titres according to instructions from the manufacturer. Microsoft Excel was also used for calculation of average titres within groups including standard deviations, graphically illustrated.

### RNA extraction and RT-PCRs

RNA extraction and the RT-PCRs were performed as previously described [25,26].

### Experimental design

Four groups (1–4), including 23, 24, 23 and 23 chickens respectively were vaccinated as recommended (Intervet, Boxmeere, The Netherlands) with strain D78 at the age of 21 days. At the age of 24 days, chickens in groups 1, 3 and 4 were inoculated orally with 1500 sporulated oocysts each. Feed was withdrawn four hours before inoculation to facilitate the flow of the oocysts into the gut. Then, 0.225 ml of the solution of sporulated oocysts was diluted in 5.775 ml tap water, and 0.2 ml of this mixture was inoculated into each chicken orally. Groups 2, 3 and 4 were subsequently challenged with vvIBDV at the age of 28 days (Table 1). Observations and registrations included clinical symptoms, pathology and serology. Three chickens from each group were euthanised on days 28 (before virus challenge), 29, 30, 31, 36, 38 and 42. On day 44, the remaining two chickens in groups 1,3 and 4, and the remaining three chickens in group 2 were sampled. Blood was collected before euthanisation, and the carcasses were subjected to autopsy immediately after death. The intestinal tract was examined for lesions due to coccidia. One half of the bursa of Fabricius, and the spleen, thymus and bone marrow were sampled and frozen at -80°C. The other half of the bursa was treated as described under histology. Bursa swabs were collected from groups 2 and 4 during post mortem examination, eluted in sterile saline for one hour and kept frozen at -20°C until analysed. All tissue samples and swab samples were initially analysed by Qiagen RT-PCR without denaturation [26]. In case of any positive results, all similar tissue samples from the same group were analysed by duplex (DPX) RT-PCR [25] for identification of the virus strain.

**Table 1:** Inoculation protocol. Number of chickens treated in each group.

	Group 1	Group 2	Group 3	Group 4
Vaccination D78, day 21	23	24	23	23
Coccidia, day 24	23		23	23
Challenge vvIBDV, day 28		21	20	20

In order to verify the presence of viable virus in the bursa tissues found positive for DK01 by DPX RT-PCR, an additional experiment was performed. A fifth group of eleven, three-weeks-old SPF chickens, bred and reared as groups 1–4 was employed. Half of each of the bursa of Fabricius found positive for DK01 was treated as previously described, before inoculation into these chickens at three weeks [26].

## Results

### Clinical signs

Chickens in groups 1–4 did not show clinical signs of disease at any time during the experimental period.

### Coccidia

Lesions were observed by gross examination of cecae from the three groups of chickens infected with coccidia (groups 1, 3 and 4) on day 31, seven days after inoculation, and at the same time numerous unsporulated oocysts, corresponding in morphology and size to *E. tenella *were seen by microscopy of scrapings from the cecae. Lesions were evaluated as score 3 in all three groups. On day 36, the 12^th ^day after inoculation, lesions consistent with lesions caused by *E. acervulina *were evaluated as score 2 in chickens from the same groups. Numerous unsporulated oocysts, corresponding in morphology and size to *E. acervulina *were seen in scrapings from duodenum and jejunum. The cecae appeared normal, and only few oocysts were observed in scrapings. Intestinal lesions were not observed after the 12^th ^day after inoculation.

Developmental forms of *E. tenella *were not observed in any tissue samples from the bursa of Fabricius.

### IBDV pathology

Gross pathological changes due to IBDV were only related to the bursa of Fabricius. Randomly occurring slight enlargements of the bursa were observed on day 28, one week after vaccination. Microscopic lesions were observed in all bursa tissue samples except one from a 38-days old chicken from group 5. The vaccine strain initially caused lymphocyte depletion, interstitial oedema and folding of follicular epithelium. After challenge with DK01, lymphocyte depletion became more pronounced, as vacuoles and necrotic cells were observed in the follicular medulla, and the structure of the follicles was dissolved. Bursa lesions observed in groups 3 and 4 included a larger part of the bursa tissue than the lesions observed in groups 1 and 2 on days 28 or 29. The lesion score decreased with time after 30 days in group 1, while the lesion scores in groups 2, 3 and 4 either remained high or increased after challenge with field virus on day 28 (Table 2). Lesion scores varied considerably within each group of three chickens.

**Table 2:** Bursa lesion score, three or two chickens sampled each time at various ages.

Age In days	Group 1	Group 2	Group 3	Group 4
28	1,4,2	1,2,3	4,4,4	3,4,4
29	4,3,2	4,2,1	4,4,4	4,4,1
30	4,1,2	2,3,4	3,4,4	1,3,3
31	1,1,1	1,1,4	3,1,4	2,4,3
36	1,2,1	1,2,1	4,1,1	2,4,1
38	1,1,1	4,4,1	1,1,4	1,1,2
42	1,1,2	1,4,2	1,4,4	3,1,4
44	1,1	2,2,2	1,1	1,2

When bursa lesions involved 1–25% of the follicles, a value of 1 was assigned, 26–50% of follicles a value of 2 was assigned, 51–75% of follicles a value of 3 was assigned, and 76–100% of follicles a value of 4 was assigned.

### Serology

Antibody titres were positive seven days after vaccination. Average titre values for each group of three chickens in several cases showed large standard deviations, and results from none of the groups deviated significantly from results from one of the other groups during the entire experiment (results not shown).

### RT-PCR

In group 1, viral RNA identified as D78 was found in bursa tissues only, 96% of these samples being positive (Table 3). In group 2, 96% of bursa samples also contained D78, and in addition, two bone marrow samples and six bursa swab samples were found positive. In groups 3 and 4, 74% and 96%, respectively, of bursa samples contained D78. Surprisingly, several spleen and thymus samples from these two groups were also found positive (Table 3). The field strain, DK01 was only detected in six samples involving four chickens out of the total of 70 chickens infected with DK01. Three of these chickens were from group 2, as DK01 was identified in bursa tissues from days 29, 31 and 44. In addition, DK01 was detected in the thymus and a bursal swab from the bursa-positive chicken from day 31. The sixth positive sample was a spleen sample from a chicken in group 3 on day 36.

**Table 3:** Vaccine virus D78. Results of DPX RT-PCR of lymphoid tissues from groups 1 to 4, and bursa swabs from groups 2 and 4.

Age	G.1				G.2					G.3				G.4				
	+ cocc, – DK01				– cocc, + DK01					+ cocc, + DK01				+ cocc, + DK01				
				
days	BF	Bm	Spl	Thy	BF	Bm	Spl	Thy	Bs	BF	Bm	Spl	Thy	BF	Bm	Spl	Thy	Bs
28	3/3	0/3	0/3	0/3	3/3	1/3	0/3	0/3	2/3	3/3	0/3	2/3	1/3	3/3	0/3	1/3	0/3	0/3
29	3/3	0/3	0/3	0/3	3/3	0/3	0/3	0/3	2/3	3/3	0/3	2/3	2/3	3/3	0/3	2/3	1/3	3/3
30	3/3	0/3	0/3	0/3	3/3	0/3	0/3	0/3	1/3	2/3	0/3	1/3	2/3	3/3	0/3	0/3	0/3	2/3
31	3/3	0/3	0/3	0/3	3/3	0/3	0/3	0/3	0/3	3/3	0/3	0/3	0/3	3/3	0/3	2/3	1/3	2/3
36	2/3	0/3	0/3	0/3	3/3	1/3	0/3	0/3	0/3	2/3	0/3	2/3	0/3	3/3	0/3	2/3	0/3	1/3
38	3/3	0/3	0/3	0/3	3/3	0/3	0/3	0/3	0/3	3/3	0/3	0/3	1/3	3/3	0/3	0/3	0/3	0/3
42	3/3	0/3	0/3	0/3	2/3	0/3	0/3	0/3	1/3	1/3	0/3	0/3	0/3	3/3	0/3	1/3	0/3	1/3
44	2/2	0/2	0/2	0/2	3/3	0/3	0/3	0/3	0/3	0/2	0/2	0/2	0/2	1/2	0/2	0/2	0/2	0/2
				
Total	22/23	0/23	0/23	0/23	23/24	2/24	0/24	0/24	6/24	17/23	0/23	7/23	6/23	22/23	0/23	8/23	2/23	9/23
%	96%	0%	0%	0%	96%	12%	0%	0%	25%	74%	0%	30%	26%	96%	0%	35%	9%	39%

G: group, BF: bursa of Fabricius, Spl: spleen, Thy: thymus, Bm: bone marrow, Bs: bursa swab, x/y: positive result/analysed samples. Total: number of positive/number of analysed, and as percentage

### Additional experiment

Two days after inoculation with bursa extract, the eleven bursa-inoculated chickens were clinically ill and consequently euthanized. Autopsy revealed swollen bursa of Fabricius and petechial muscular bleedings. DPX RT-PCR using tissue from the bursa of Fabricius detected both D78 and DK01 in all bursa samples (data not shown).

## Discussion

Although clinical symptoms were not observed, lesion scores and microscopy documented subclinical coccidiosis in groups 1, 3 and 4 two days after the chickens in groups 3 and 4 were challenged with field virus. From the same day, unprotected chickens would be expected to show clinical IBD symptoms [26]. The presence of subclinical coccidiosis did not have a negative impact on the vaccination against IBD significant enough to provoke clinical symptoms, leaving the reasons for vaccine breaks observed under field conditions unexplained. The reasons why we did not observe an aggravating effect on the immune system favouring *E. tenella *as previously reported [14] could be differences in IBDV strains, differences in doses of sporulated coccidia (1500/150 000), or that our experimental inoculation with coccidia only resulted in a single life cycle in the host, probably due to the wire floor in the isolators. Different ages of the chickens also may have influenced the experiments.

Bursa lesion scores were more severe for groups 3 and 4 than for group 2, except for group 4 on day 30 and both groups on days 38 and 44. The presence of coccidia might have aggravated the development of lesions in the bursa caused by IBDV. As the number of samples in each age group was too small for reliable statistical evaluation of results, further investigations are needed for documentation of a potential influence of coccidia on bursa lesions caused by IBDV.

Serology was used only in order to confirm, that all chickens had seroconverted. Titre values were not further interpreted, as this would require investigations into the technical performance of the ELISA-kit used, falling outside the scope of this work [31].

D78 was detected in spleen and thymus tissues from groups 3 and 4 but not from group 2, indicating that the presence of coccidia may have influenced the distribution of vaccine strain RNA. However, this theory was impaired by the lack of D78 in the spleen and thymus tissues from group 1. *Zhang et al*. [32] detected a cell adapted IBDV strain in spleen and thymus tissues four hours *p.i*. We speculated that this could indicate that vaccine strains generalize and replicate continuously in various lymphoid tissues, but at a lower level than in the bursa, and thus mostly undetectable by our current methods, until replication was enhanced in groups 3 and 4. As enhanced replication in the spleen and thymus was not recorded in groups 1 and 2, the present reaction seemed to depend on a concurrent stimulation of both coccidia and vvIBDV. *Rautenschlein et al*. [33] suggested that systemic antigen stimulation caused by enhanced replication of IBDV in extrabursal tissues may result in improved IBD protection. This may explain that we only detected vvIBDV in a single spleen sample but not in the bursa tissues or bursal swabs of groups 3 and 4, not indicating any excretion of vvIBDV in these groups. In comparison, we showed replication of DK01 in bursa tissues from three chickens in the non-coccidia-inoculated group 2, suggesting excretion from at least one of them, detected as presence of viral RNA in the bursa swab. Indications of replication and excretion of vvIBDV in vaccinated chickens have been discussed in a previous paper [25]. The significance of the D78-positive bone marrow samples from group 2 remains to be investigated.

## Conclusion

In conclusion, coccidia did not seem to affect IBDV vaccination in chickens negatively. On the contrary, our results suggested an additive effect of concurrent stimulation of the immune system by subclinical coccidiosis and vvIBDV, enhancing the replication and distribution of the vaccine strain in chicken lymphoid tissues. Assuming that replication of and presence of vaccine virus in extra-bursal lymphoid tissues mediates improved protection, our experiment indicated that coccidia contributed to an improved immune response following IBDV vaccination. The perspectives of these conclusions might be a possibility of benefiting from an enhancing immunological effect of concurrent, controlled viral and parasitic infections. Further research into immunological consequences of complex infections in chickens is highly relevant.

## Competing interests

The author(s) declare that they have no competing interests.

## Authors' contributions

SK designed and carried out the experiments and drafted the manuscript

KJH supervised and adjusted laboratory processes

MB conceived of the study and participated in drafting the manuscript

All authors read and approved the final manuscript
